# A mechanism-based pharmacokinetic model of fenofibrate for explaining increased drug absorption after food consumption

**DOI:** 10.1186/s40360-018-0194-5

**Published:** 2018-01-25

**Authors:** Hyun-moon Back, Byungjeong Song, Sudeep Pradhan, Jung-woo Chae, Nayoung Han, Wonku Kang, Min Jung Chang, Jiao Zheng, Kwang-il Kwon, Mats O. Karlsson, Hwi-yeol Yun

**Affiliations:** 1 College of pharmacy, Chungnam National University, 99 Daehak-ro, Yuseong-gu, Daejeon, 34134 South Korea; 2 Drug discovery center, JW Pharmaceutical, Seoul, 06725 South Korea; 3 School of Pharmacy, University of Otago, 56, Dunedin, 9054 New Zealand; 4 College of Pharmacy, Seoul National University, Seoul, 03080 South Korea; 5 College of pharmacy, Chung-Ang University, Seoul, 06974 South Korea; 6 College of Pharmacy and Yonsei Institute of Pharmaceutical Sciences, Yonsei University, Incheon, 21983 South Korea; 7 Department of Pharmaceutical Medicine and Regulatory Science, College of Medicine and Pharmacy, Yonsei University, Incheon, 21983 South Korea; 8 Department of Pharmacy, Huashan Hospital, Fudan University, Shanghai, 200040 China; 9 Department of Pharmaceutical Biosciences, Uppsala University, 75124 Uppsala, Sweden

**Keywords:** Food effect, Gastrointestinal system, Fenofibrate, NONMEM, Drug absorption

## Abstract

**Background:**

Oral administration of drugs is convenient and shows good compliance but it can be affected by many factors in the gastrointestinal (GI) system. Consumption of food is one of the major factors affecting the GI system and consequently the absorption of drugs. The aim of this study was to develop a mechanistic GI absorption model for explaining the effect of food on fenofibrate pharmacokinetics (PK), focusing on the food type and calorie content.

**Methods:**

Clinical data from a fenofibrate PK study involving three different conditions (fasting, standard meals and high-fat meals) were used. The model was developed by nonlinear mixed effect modeling method. Both linear and nonlinear effects were evaluated to explain the impact of food intake on drug absorption. Similarly, to explain changes in gastric emptying time for the drug due to food effects was evaluated.

**Results:**

The gastric emptying rate increased by 61.7% during the first 6.94 h after food consumption. Increased calories in the duodenum increased the absorption rate constant of the drug in fed conditions (standard meal = 16.5%, high-fat meal = 21.8%) compared with fasted condition. The final model displayed good prediction power and precision.

**Conclusions:**

A mechanistic GI absorption model for quantitatively evaluating the effects of food on fenofibrate absorption was successfully developed, and acceptable parameters were obtained. The mechanism-based PK model of fenofibrate can quantify the effects of food on drug absorption by food type and calorie content.

**Electronic supplementary material:**

The online version of this article (10.1186/s40360-018-0194-5) contains supplementary material, which is available to authorized users.

## Background

Oral administration of drugs has several advantages over other administration routes, including lack of pain, ease of administration, portability and availability of convenient dosage forms (dissolving tablets, sustained-release (SR) tablets, and solution or suspension forms), which increases compliance in patients [1] . However, oral drug administration has certain limitations compared with intravenous (IV) administration that are mainly due to the influence of physiological factors in the gastrointestinal (GI) system, such as gastric acid secretions, pH, gastric emptying rate, secretion of bile acid and food consumption [2]. Among these physiological conditions, food consumption is a major factor that can affect drug absorption by influencing the entire GI system through increased gastric acid secretion, gastric emptying rate and bile acid production. Consequently, absolute drug absorption is affected by changes in GI system conditions, meaning that systemic circulation absorption of drugs can vary even when the same dose is given. Therefore, many researchers are attempting to define how food-induced changes in the GI system affect drug absorption using mathematical modelling techniques, such as a population analysis, and physiologically based pharmacokinetic (PBPK) modelling.

Most previous mathematical modelling studies on the effects of food considered food as a categorical covariate [3–5], but this appears to be insufficient to explain changes observed in the physiological condition of the GI system in response to food type and calorie content. Another approach for determining the effect of food on absorption is via PBPK modelling [6, 7], but this approach requires a large amount of information and quantification of physiological conditions, making it highly complex with little flexibility. Therefore, it would be useful to explain the effects of food-drug interactions on drug absorption by building a simple and powerful mechanistic model for predicting the effects of food while considering the physiological conditions.

Fenofibrate is a well-known drug to be affected by food consumption on absorption. In addition, it is rapidly metabolised by esterase to fenofibric acid, the main active metabolite in the blood, which is excreted in urine along with fenofibric acid glucuronide [8]. A previous report showed that absorption of fenofibric acid was increased roughly 35% in fed conditions compared with fasted conditions [9]. Another study reported that the maximum plasma concentration (*C*_*max*_) and the area under the plasma concentration-time curve (*AUC*_*inf*_) for fenofibric acid were significantly increased when the drug was administered via a SR capsule immediately after food consumption [3].

Despite the significant effects of GI tract condition on the absorption of fenofibrate, there have been no reports on the quantitative assessment of food effects on fenofibrate absorption. Therefore, this study aimed to develop a mechanism-based pharmacokinetic (MBPK) model for quantifying the influence of food-fenofibrate interactions on drug absorption. Multiple doses of fenofibrate under fasted and fed conditions were simulated to evaluate accumulation effects of food consumption on systemic exposure of fenofibrate.

## Methods

### Collection of food-fenofibrate interaction data

An initial clinical screening process (physical examination and laboratory tests) was performed, and 24 healthy Korean subjects (13 males, 11 females) were selected to participate in the randomized, three-way crossover trial with a single oral dose of a 250 mg fenofibrate (SR) capsule and three different food types (Additional file 1). The study protocol was approved by the Food and Drug Administration of Korea, and the Ethics Committee of the Institute of Drug Research and Development at Chungnam National University. Written informed consent was obtained from all participants. The subjects received a single 250 mg fenofibrate SR capsule with 240 mL of water 10 min after consuming no food, a standard breakfast or a high-fat breakfast. The composition of meals is shown in Table 1. Meal composition (fat and calorie content) was chosen based on US Food and Drug Administration (FDA) and Ministry of Food and Drug Safety (MFDS) in Korea guidance for food-effect bioavailability and fed bioequivalence studies [10, 11], and meal type was adapted according to MFDS recommendations and standard Korean nutrition habits. All subjects received the same lunch and supper. The design and results of this study were reported previously [3].

**Table 1:** Calorie, nutrients content ratio and food content in standard meal and high fat meal which were given to participants of study before administration of the drug

Food type group	Fasting condition	Standard meal	High fat meal
Calories	0 kcal	686.3 kcal	1280 kcal
Nutrients content ratio	–	Carbohydrate (56.3%)	Carbohydrate (45.5%)
		Protein (23.9%)	Protein (19%)
		Fat (19.9%)	Fat (35.5%)
Food contents	–	Steamed rice (90 g)	Steamed rice (90 g)
		Soup (shrimp dried (20 g), sea mustard dried (5 g))	Soup (sea mustard dried (6 g), beef brisket (30 g))
		Grilled yellow croaker (70 g)	Fried yellow croaker (70 g) with Soybean oil (5 g)
		Steamed whole egg (50 g) with sesame oil (1 g)	Egg roll (whole egg (50 g) with soybean oil (3 g))
		Korean radish egg (70 g)	Boiled spinach (70 g) with sesame oil (1 g) and soybean oil (1 g)
		Kimchi (60 g)	Kimchi (60 g)
		Apple (100 g)	Cracker with peanuts (32 g)

### Pharmacokinetic modeling

Development of the MBPK model was performed using NONMEM (version 7.3; ICON). The first-order conditional estimation with interaction (FOCE-I) method was used for parameter estimation. Fenofibric acid, the active form of fenofibrate, was used as a representative of fenofibrate for PK profiling due to low systemic exposure resulting from rapid metabolism in the GI tract and blood by esterases.

Fenofibric acid PK data and the calorie content of two different food types were modelled using five physiological compartments and parameters modified from a previous study [12]. The differential equations used to describe fenofibric acid PK data and calorie content in physiological compartments were as follows:

$$ \frac{dX1}{dt}=-{k_g}^{\ast }X1 $$

$$ \frac{dX2}{dt}={k_g}^{\ast }X1-{k_{m\&a}}^{\ast}\left(1+{E_{bile}}^{\ast }X5\right)\ast X2 $$

$$ \frac{dX3}{dt}={k_{m\&a}}^{\ast}\left(1+{E_{bile}}^{\ast }X5\right)\ast X2-{k_{el}}^{\ast }X3 $$

$$ \frac{dX4}{dt}=-{k_g}^{\hbox{'}}\ast X4 $$

$$ \frac{dX5}{dt}={k_g}^{\hbox{'}}\ast X4-{k_{out}}^{\ast }X5 $$

Where *X*_*i*_ (*i* = 1, 2, 3) is the amount of drug in each compartment, *X*_*j*_ (*j* = 4, 5) is the number of calories in each compartment, and *k*_*g*_ and *k*_*g*_*’* are the gastric emptying rate constant of the drug and the number of calories in the GI tract carried from the stomach to the duodenum, respectively. The *k*_*m&a*_ value represents the mixed rate constant of the rate of metabolism from fenofibrate to fenofibric acid, and the absorption rate of fenofibric acid from the duodenum-upper intestine compartment to the central compartment. The rate of fenofibrate metabolism and the absorption rate of fenofibric acid should not be separated due to the characteristics of fenofibrate, which is metabolized completely and rapidly convert to fenofibric acid in the body by esterases. *K*_*el*_ and *k*_*out*_ are the elimination rate constant for fenofibric acid from the central compartment, and the number of calories in the duodenum-upper intestine compartment, respectively. *E*_*bile*_ represents the effect of bile acid on the fenofibrate metabolic rate and the fenofibric acid absorption rate constant. To evaluate the effect of bile, linear and nonlinear (Michaelis-Menten) equations were tested and selected based on graphical (goodness-of-fit plot) and numerical (objective function value) diagnostics.

The time-varying gastric emptying rate was included in the model as follows:

$$ {\mathrm{k}}_{g\_i}={TVk}_g\cdot {\left(1+{E}_{food}\right)}^{\left(\mathrm{mpast}(1)-\mathrm{mpast}(2)\right)}\cdot {e}^{\eta_i} $$

where *k*_*g_i*_ is the gastric emptying rate of the *i*^th^ individual, *TVk*_*g*_ is the typical value (TV) of the fasted gastric emptying rate and *E*_*food*_ represents the coefficient for quantifying the effect on gastric emptying rate after food consumption. If an individual is under fasting conditions, *k*_*g_i*_ is estimated as *TVk*_*g*_ alone, where *E*_*food*_ is calculated in fed conditions only. MPAST and MTIME were used as equation options in NONMEM for explaining changes in parameter values over time. MPAST(i) is 0 until MTIME(i), but changes to 1 when MTIME(i) and MTIME are even, as estimated by the model. In the above equation, MPAST(1) has a constant value of 1 when MTIME(1) is fixed at 0. However, MPAST(2) will be equal to 0 before MTIME(2), and changes to 1 after MTIME(2) [13]. Thus, MPAST(1) - MPAST(2) will be equal to 1 during the time between MTIME(1) and MTIME(2), and after MTIME(2) mpast will change to 0. These equations allow calculation of the effect of food on the gastric emptying rate during digestion.

The effect of food on the central volume of distribution is obtained by adding E_*Vc*1_ and E_*Vc*2_ as follows:

$$ {TVV}_{c/F\_1}={\uptheta}_i $$

$$ {TVV}_{c/F\_2}={\uptheta_i}^{\ast}\left(1+{E}_{Vc1}\right) $$

$$ {TVV}_{c/F\_3}={\uptheta_i}^{\ast}\left(1+{E}_{Vc2}\right) $$

where θ_*i*_ is the typical apparent volume of the distribution, and E_*Vc*1_ and E_*Vc*2_ represent coefficients for quantifying the effect of food consumption on bioavailability. Under fasting conditions, *TV*_*Vc*/*F*_ is estimated as θ_*i*_ alone, where *E*_*Vc*1_ and E_*Vc*2_ are calculated in fed conditions (*E*_*Vc*1_ = standard meal, E_*Vc*2_ = high-fat meal) only.

The exponential error model $\left({e}^{\eta_i}\right)$ was used to explain the inter-individual variability (IIV) of the gastric emptying rate, *V*_*c*_*/F* (Apparent central volume of distribution of fenofibic acid), *k*_*el*_ and also inter occasion variability (IOV) [14] was also considered for *V*_*c*_*/F* and *k*_*el*_. The proportional error model was used to explain the residual variability of the model. During the development of the model, the OFV of each nested model was compared (delta OFV > 3.84) to determine the statistical significance (*p* < 0.05).

### Model evaluation

Goodness-of-fit plots [15] and visual predictive checks (VPCs) were performed to assess model development and the final model. A VPC [16] was performed with 1000 simulated samples from the final model to evaluate the prediction properties by perl speaks nonmem ver.4.6.0. Estimated relative standard error (RSE) and non-parametric bootstrap methods were implemented to evaluate the precision of the final model estimates [17]. Random sampling was performed by extracting 90% of data from the original dataset and generating new datasets for internal validation. Generated datasets were then used to obtain new PK parameters with the final model, and the process was replicated 1000 times to calculate confidence intervals for each PK parameter. Finally, the median value and the 95% confidence interval of parameters obtained from the bootstrap analysis were compared with parameters from the final model [18].

### Simulation

Simulation of three different scenarios (Table 2) was performed using the final model with 1000 individuals in each group receiving once daily administration of a 250 mg fenofibrate SR capsule for 7 days. Each group (fasted, normal meal and high-fat meal) received the corresponding meal type before taking the drug on each of the 7 days. To compare the accumulation effects of food on systemic exposure of fenofibrate acid, the AUC_168➔192_ (steady state) and C_max_ss_ of each group at steady state was used. Statistical significance was assessed using analysis of variance (ANOVA) performed in SPSS software (version 22; IBM).

**Table 2:** Simulation scenarios for final mechanism-based PK model of fenofibrate (n = 1000)

	Group 1	Group 2	Group 3
Calories	0 kcal	400 kcal	1280 kcal
Food type	–	Normal meal (Low fat)	High fat meal
Diet	–	Banana × 3	Hamburger (236 g)
		Boiled Egg × 2	Fried potatoes (114 g)
			Beverage (425 mL)

## Results

### Data collection

This study was carried out between April 2002 and November 2002. Demographic data gathered from the subjects involved in the study were generally similar; all subjects were Korean (13 male, 11 female) with a mean age of 23 years, a mean weight of 68.75 kg and a mean height of 173.29 cm (Additional file 2). Blood samples were collected before and at 1, 2, 3, 4, 5, 6, 8, 10, 12, 24, 48 and 72 h after drug administration. The PK profiles of fenofibric acid after administration of a 250 mg fenofibrate SR capsule in fasted and different meal groups are shown in Additional file 3.

### Pharmacokinetic model

**Fig. 1:**
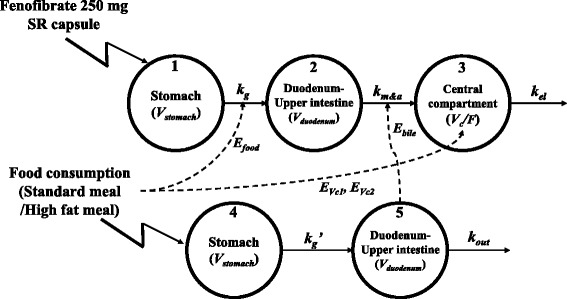
Scheme showing the mechanism-based pharmacokinetic model for explaining increased fenofibrate absorption after food consumption. (V_stomach_ and V_duodenum_: Volume of distribution in the stomach and duodenum, *k*_*g*_ (*k*_*g*_*’*) and *k*_*out*_: Gastric emptying rate constant and the elimination rate constant for calories, *k*_*m&a*_: Metabolism and absorption rate constant of fenofibrate, *k*_*el*_: Elimination rate constant, V_c_/F: Volume of distribution, E_food_, E_bile_, E_Vc1_ and E_Vc2_: Parameters for explaining the effects of food on fenofibrate absorption)

The final MBPK model consisted of a three-compartment model for fenofibrate and fenofibric acid, and a two-compartment model for food consumption (Fig. 1). Estimated parameters for the final MBPK model are shown in Table 3. After fenofibrate entered the stomach compartment, the gastric emptying rate constant to the duodenum compartment (*k*_*g*_) was 0.0412 h^− 1^, and the combined rate of metabolism of fenofibrate and absorption of fenofibric acid from the duodenum to the central compartment (*k*_*m&a*_) was 0.198 h^− 1^. The volume of distribution of fenofibric acid (V_c_/F) was 12.9 L, and the elimination rate constant of fenofibric acid (*k*_*el*_) was 0.27 h^− 1^. Stomach volume was fixed as 49 mL and 1 L under fasting and fed conditions, respectively [19]. The gastric emptying rate constant for food from the stomach to the duodenum (*k*_*g*_*’*) was 0.00971 h^− 1^, and the calorie elimination rate constant (*k*_*out*_) was 0.00972 h^− 1^. The duration of food effect (MTIME) was 6.94 h, and the effect on the gastric emptying rate (*E*_*food*_) was 0.617. The effect of bile acid on the absorption rate constant of fenofibric acid was 0.0239, and the effects on volume of distribution by food type (E_*Vc*1_, E_*Vc*2_) were − 0.394 and − 0.461, respectively.

**Table 3:** Estimated parameters from the final MBPK model to evaluate effect of food on fenofibrate absorption and results from bootstrap

Parameters	Estimates (% RSE)	IIV %CV (% RSE)	IOV %CV (% RSE)	Bootstrap median ± S.E.
*k*_*g*_ (hr^−1^)	0.0412 (8.5%)	31.7% (22.1%)	–	0.042 ± 0.0033
*k*_*m&a*_ (hr^− 1^)	0.198 (28.9%)	–	–	0.172 ± 0.057
*k*_*el*_ (hr^− 1^)	0.27 (13. 6%)	86.3% (29.2%)	44.9% (43.5%)	0.28 ± 0.048
*V*_*c*_*/F* (L)	12.9 (27.8%)	93% (28.9%)	50.9% (22.6%)	11.91 ± 2.55
*V*_*stomach*_ (L)	0.049^a^/1^a^	–	–	0.049^a^/1^a^
*V*_*duodenum*_ (L)	0.045^a^	–	–	0.045^a^
*k*_*g*_*’* (hr^−1^)	0.00971 (58.9%)	–	–	0.011 ± 0.005
*k*_*out*_ (hr^−1^)	0.00972 (38.3%)	–	–	0.011 ± 0.005
*E*_*bile*_	0.0239 (22.2%)	–	–	0.027 ± 0.091
MTIME1 (hr)	0^a^	–	–	0^a^
MTIME2 (hr)	6.94 (12.4%)	–	–	6.92 ± 1.11
*E*_*food*_	0.617 (40.4%)	–	–	0.64 ± 0.19
*E*_*Vc1*_	−0.394 (40.1%)	–	–	− 0.38 ± 0.12
*E*_*Vc2*_	−0.461 (37.7%)	–	–	− 0.43 ± 0.14
Residual variability				
Proportional error (% RSE)	0.608 (6.4%)			0.599 ± 0.038

^a^Fixed parameter

The IIV of *k*_*g*_ (*k*_*g*_*’*, shared IIV), *Vc/F* and *k*_*el*_ was 31.7%, 93 and 86.3%, respectively. The IOV of *Vc/F* and *k*_*el*_ was 44.9 and 50.9%, respectively, and the residual error was 60.8%.

### Model evaluation

The goodness of fit of the final model was evaluated by log-transformed observation (DV) versus log-transformed population prediction (PRED), individual prediction (IPRED) plot and a conditional weighted residual (CWRES) versus time plot. Log-transformed individual prediction values matched well with log-transformed observation values, and displayed linearity. An unbiased conditional weighted residual versus time plot is shown in Additional file 4.

**Fig. 2:**
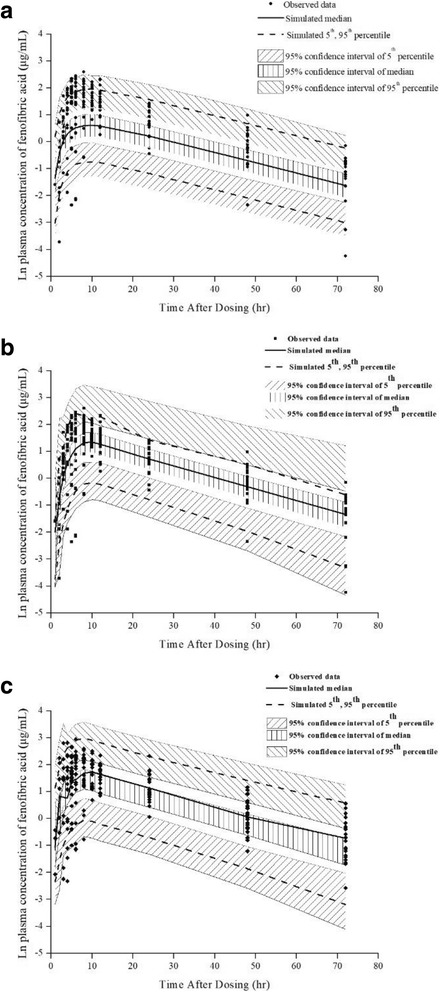
Visual predictive check plots for the final MBPK model (*n* = 2000). (**a** fasting conditions, **b** standard meal, **c** high-fat meal)

The precision of the final estimated parameters was assessed via the RSE (%) of the final model and bootstrapping. The median value and the standard error of the parameters are shown in Table 3. The prediction power of the final model was assessed via a VPC. The predicted 95% confidence intervals of the 5th, 50th and 95th percentiles covered the observed data well for corresponding meal types (Fig. 2). The VPC results showed that the plasma concentrations of fenofibric acid were successfully explained by the final model, including the effect of food in the corresponding meal types. The bootstrap confirmed the robustness and stability of the final model and the estimated parameters.

### Simulation

**Fig. 3:**
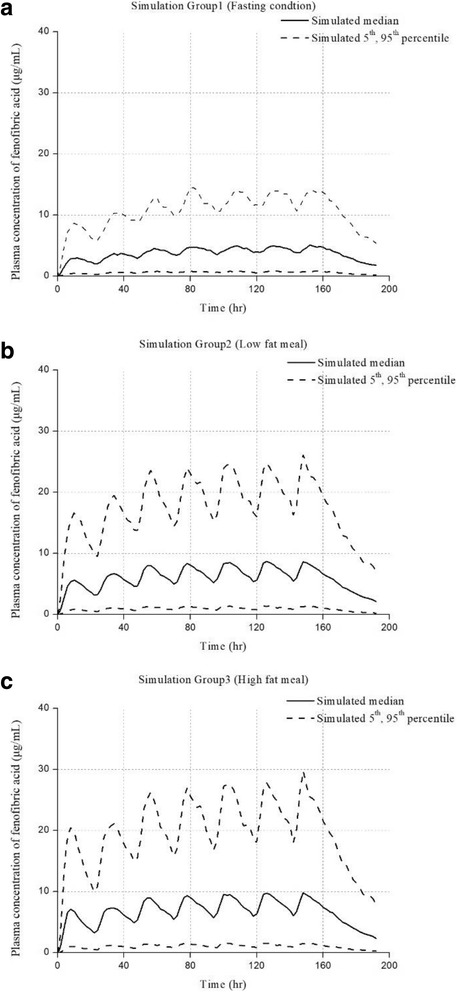
Simulated plasma concentrations of fenofibric acid (*n* = 1000). (**a** fasting conditions, **b** standard meal, **c** high-fat meal)

To predict the exposure of fenofibric acid following different meal types, simulations were performed using the final model with three scenarios. The purpose of this simulation was to evaluate differences in drug exposure caused by different meal types when fenofibrate was taken orally. Plasma concentration plots of fenofibric acid after administration of a 250 mg fenofibrate SR capsule for 7 days with each meal type are shown in Fig. 3. The AUC_168➔192_ and C_max_ of fenofibric acid after administration of the last dose of fenofibrate on Day 7 are shown in Table 4. ANOVA performed on AUC_168➔192_ and C_max_ values calculated from the simulated data showed that both parameters were significantly increased under fed conditions compared with fasting condition.

**Table 4:** C_max_ and AUC_168➔192_ of fenofibric acid at steady state from simulated data using final MBPK model and results of ANOVA

Mean ± S.E.	Group 1	Group 2	Group 3
Food type	Fasting condition	Normal meal	High fat meal
C_max_ (**μ** g/mL)	3.17 ± 0.07	4.33 ± 0.10^*^	4.83 ± 0.11^*†^
AUC_168➔192_ (**μ** g∙hr./mL)	319.14 ± 7.48	432.90 ± 10.33^*^	483.63 ± 11.55^*†^

^*^: *p* < 0.01 vs the Group 1; ^†^: p < 0.01 vs the Group 2

## Discussion

In this study, we developed a MBPK model to quantitatively evaluate changes in the physiological conditions in the GI tract after food consumption, and the effect this has on the fenofibrate PK profile. The final model consisted of five compartments, including four physiological compartments and one central compartment for explaining systemic exposure of the drug. The volume of the stomach was generally altered by food consumption during digestion, and this change affected the distribution of the drug inside the stomach. To describe this effect after food consumption, we fixed physiological parameters with literature values where available. In the same manner, the duodenum-upper intestine compartment was given a fixed value of 0.045 L [19]. Although 0.045 L is a relatively small value for the volume of the duodenum-upper intestine, it is suitable given that most of drug was absorbed from the duodenum to a local site in the upper intestine.

To evaluate the incremental gastric emptying rate (k_g_) after food consumption, and its duration, MTIME and *E*_*food*_ were estimated. The duration for which the gastric emptying rate was affected after food consumption was 6.94 h, and the gastric emptying rate was increased by 61.7% (Table 5). These results are in good agreement with a previous report on the digestion time of food. When food passes through the duodenum after consumption, bile acid secretion is stimulated to assist with digestion, and this increase in bile acid secretion can affect the absorption of drugs because its chemical characteristics ensure that it binds to and solubilises lipophilic drugs. To describe the above conditions, a linear equation for explaining the effect of bile acid (*E*_*bile*_) on drug absorption was included in the final model. The change in the number of calories in the duodenum-upper intestine compartment was directly proportional to absorption (k_m&a_) of the drug (Table 5), in accordance with the change in the rate of bile acid secretion proportional to the amount of food in the duodenum.

**Table 5:** Increased (or decreased) pharmacokinetic parameters values and percent change of fenofibrate after consumption of food, relative to fasted state

	Standard meal (686.3 kcal)	High fat meal (908 kcal)
*k*_*g*_	0.067 h^− 1^ (+ 61.7%)	
*k*_*m&a*_	0.23 h^− 1^ (+ 16.5%)	0.24 h^− 1^ (+ 21.8%)
V_c_/F	7.82 L (− 39.4%)	6.95 L (− 46.1%)

Since drug bioavailability is most likely affected by food type such as standard or high-fat meal content, the effect of food type on *Vc/F* was evaluated. *Vc/F* values estimated from the model were lower in standard and high-fat meal groups than the fasted group. Because drugs are affected by food intake, bioavailability can be altered, but the location of distribution is generally unaffected. This aspect can be explained by the increase in *F* (bioavailability) with apparent *Vc*. Therefore, the bioavailability of fenofibric acid increased 1.65- or 1.86-fold after ingestion of a standard or high-fat meal, respectively, compared with fasting conditions.

A simulation study was conducted to determine differences in drug accumulation exposure after ingesting the same meal types before drug administration. The observed increase in systemic exposure of fenofibrate following food consumption clearly indicates that taking fenofibrate with a large amount of high calorie food can lead to significant increases in fenofibric acid exposure, and this can potentially lead not only to more efficient treatment of diseases such as dyslipidaemia, but also to an increased incidence of adverse effects like myopathy.

Conventional research on food effects is often carried out by developing a PK model with categorised parameter values to explain PK differences between fasting and fed conditions. Such PK models are generally easy to fit, but they are not able to consider physiological conditions. By contrast, the model developed in the present study is able to consider physiological conditions in the GI tract, and can therefore explain the effects of food consumption on drug absorption using mechanism-based modelling. The model not only explains differences between fasting and fed conditions (standard meal), but also compared PK profiles in the high-fat group. The model successfully explained exposure of fenofibrate in three different scenarios (fasting conditions, standard meals and high-fat meals). The final model will be tested using other drugs in the future to determine if it can be applied generally to assess the relationship between the physiology of the GI tract and the mechanism of drug absorption. Finally, our approach could significantly advance PK model development to better understand the effects of food intake on drug absorption and exposure.

## Conclusions

In conclusion, a robust MBPK model for fenofibrate incorporating the effects of food intake was successfully developed. This final model has limitations because it was developed using data from healthy individuals rather than diseased patients, and it considered food type in terms of calories alone, rather than other factors such as food volume. Despite the above limitations, this study provides a comprehensive analysis of the effects of food intake on the PK of an example drug lacking detailed PBPK information. Additionally, the effects of food on drug absorption can be quantified based on the amount of calories and the type of food (standard or high-fat meals) using the final model.

## Additional files

Additional file 1: Randomized, three-way crossover trial design with a single oral dose of a 250 mg SR fenofibrate capsule and three different food types. (DOCX 56 kb)

Additional file 2: Demographic characteristics of study participants. (DOCX 14 kb)

Additional file 3: Pharmacokinetic profile of fenofibric acid after adiminstration of a 250 mg SR fenofibrate capsule in three different meal groups. Closed circle = fasting condition; closed squared = standard meals; closed triangles = high fat meals. (DOCX 4320 kb)

Additional file 4: Goodness of fit plot of final MBPK model. (a) Natural log transformed individual predicted concentration (μ g/mL) versus natural log transformed observed concentration of fenofibric acid (μ g/mL). (b) Time (hr) versus conditional weighted residuals. Open circles: observed data points; line: least-squares regression line. (DOCX 8627 kb)
