# Supplementary material for: A mechanism-based pharmacokinetic model of fenofibrate for explaining increased drug absorption after food consumption
Source: BMC Pharmacol Toxicol. 2018 Jan 25;19:4. doi: 10.1186/s40360-018-0194-5 (PMC5785874; doi:10.1186/s40360-018-0194-5)
Supplement: Supplementary file 1 — Randomized, three-way crossover trial design with a single oral dose of a 250 mg SR fenofibrate capsule and three different food types. (DOCX 56 kb) [file 40360_2018_194_MOESM1_ESM.docx]

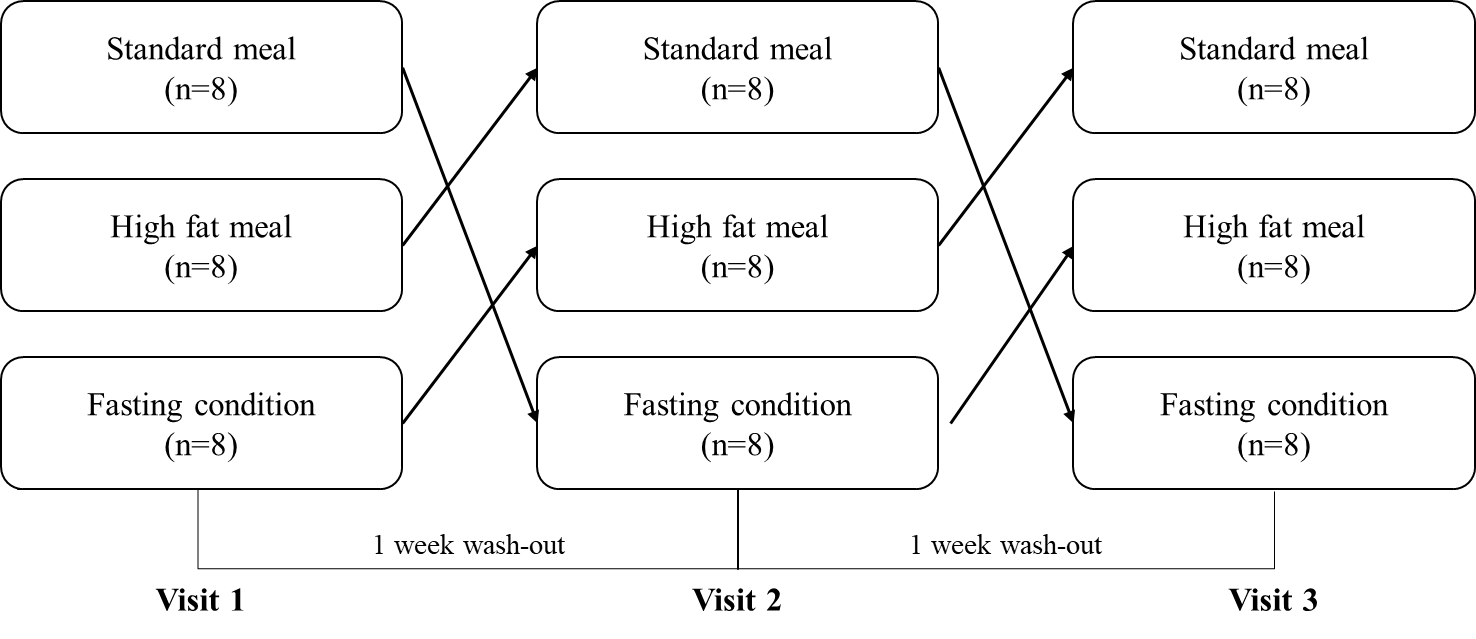


Supplementary 1. Randomised, three-way crossover trial design with a single oral dose of a 250 mg SR fenofibrate capsule and three different food types.
