# Supplementary material for: A mechanism-based pharmacokinetic model of fenofibrate for explaining increased drug absorption after food consumption
Source: BMC Pharmacol Toxicol. 2018 Jan 25;19:4. doi: 10.1186/s40360-018-0194-5 (PMC5785874; doi:10.1186/s40360-018-0194-5)
Supplement: Supplementary file 2 — Demographic characteristics of study participants. (DOCX 14 kb) [file 40360_2018_194_MOESM2_ESM.docx]

Supplement 2. Demographic characteristics of study participants.

| **Demographic data** | **Mean**$\boldsymbol{\pm}$**S.D.** |
| --- | --- |
| Sex | 13 (male) : 11 (female) |
| Age (year) | 23 (21-28) |
| Weight (kg) | 68.75$\pm$6.60 |
| Height (cm) | 173.29$\boldsymbol{\pm}$5.62 |
| Serum creatinine (mg/dL) | 0.9$\boldsymbol{\pm}0.2$ |
| AST (IU/L) | 18.7$\pm$4.9 |
| ALT (IU/L) | 15.4$\pm$7.7 |
| Total cholesterol (mg/dL) | 175.9$\boldsymbol{\pm}$22.0 |
| Blood urea nitrogen (mg/dL) | 14.5$\boldsymbol{\pm}$15.3 |
| Total bilirubin (mg/dL) | 0.8$\boldsymbol{\pm}$0.3 |
