# Supplementary material for: A mechanism-based pharmacokinetic model of fenofibrate for explaining increased drug absorption after food consumption
Source: BMC Pharmacol Toxicol. 2018 Jan 25;19:4. doi: 10.1186/s40360-018-0194-5 (PMC5785874; doi:10.1186/s40360-018-0194-5)
Supplement: Supplementary file 3 — Pharmacokinetic profile of fenofibric acid after adiminstration of a 250 mg SR fenofibrate capsule in three different meal groups. Closed circle = fasting condition; closed squared = standard meals; closed triangles = high fat meals. (DOCX 4320 kb) [file 40360_2018_194_MOESM3_ESM.docx]

Supplementary 3. Pharmacokinetic profile of fenofibric acid after administration of a 250 mg SR fenofibrate capsule in three different meal groups. Closed circles = fasting conditions; closed squares = standard meals; closed triangles = high-fat meals.
