# Supplementary material for: A mechanism-based pharmacokinetic model of fenofibrate for explaining increased drug absorption after food consumption
Source: BMC Pharmacol Toxicol. 2018 Jan 25;19:4. doi: 10.1186/s40360-018-0194-5 (PMC5785874; doi:10.1186/s40360-018-0194-5)
Supplement: Supplementary file 4 — Goodness of fit plot of final MBPK model. (a) Natural log transformed individual predicted concentration (μ g/mL) versus natural log transformed observed concentration of fenofibric acid (μ g/mL). (b) Time (hr) versus conditional weighted residuals. Open circles: observed data points; line: least-squares regression line. (DOCX 8627 kb) [file 40360_2018_194_MOESM4_ESM.docx]

Supplement 4. Goodness of fit plot of final MBPK model. (a) Natural log transformed individual predicted concentration ($\mu$g/mL) versus natural log transformed observed concentration of fenofibric acid ($\mu$g/mL). (b) Time (hr) versus conditional weighted residuals. Open circles: observed data points; line: least-squares regression line.
